# Effectiveness of pharmacotherapies for diabetes on nicotine, food, and water intake in insulin-resistant rats

**DOI:** 10.3389/adar.2023.11812

**Published:** 2024-01-04

**Authors:** Sebastian Ortegon, Priscilla Giner, Bryan Cruz, Luis M. Carcoba, Benjamin Clapp, Deborah J. Clegg, Laura E. O’Dell

**Affiliations:** ^1^ Department of Psychology, The University of Texas at El Paso, El Paso, TX, United States; ^2^ Department of Molecular Medicine, The Scripps Research Institute, La Jolla, CA, United States; ^3^ Texas Tech University Health Science Center-El Paso, Paul Foster School of Medicine, El Paso, TX, United States

**Keywords:** sex differences, insulin resistance, insulin, tobacco, high fat diet

## Abstract

The intersectionality between diabetes medications and nicotine consumption was assessed in female and male rats. Briefly, the rats were fed a high-fat diet (HFD) or regular diet (RD) for 4 weeks. Then separate groups received vehicle or a low dose of streptozotocin (STZ; 25 mg/kg). Three days later, insulin resistance was assessed by measuring plasma glucose levels for 180 min following an injection of insulin (0.75 U/kg). The rats were then prepared with jugular catheters, and they were given 23 h access to nicotine intravenous self-administration (IVSA) in 4 days cycles with 3 days of forced abstinence in their home cages where they consumed their respective diet. During the IVSA sessions, operant responses for food and water and changes in body weight were recorded. Prior to administration of the pharmacotherapies, the rats were given access to two doses of nicotine (0.015 then 0.03 mg/kg for the remainder of the study). Then, daily injections of the pharmacotherapies were given at the onset of dark cycle (6 p.m.) in the following order: 1) dapagliflozin (3.0 then 10.0 mg/kg), 2) insulin (0.75 U/kg twice), and 3) bromocriptine (3.0 then 10.0 mg/kg). The results suggest that our HFD+STZ regiment induced insulin resistance in female and male rats. Also, the HFD-fed rats displayed higher nicotine intake than RD controls, regardless of sex. Administration of insulin, but not dapagliflozin or bromocriptine, normalized nicotine intake in HFD-fed rats to control levels. These results have clinical implications regarding the potential efficacy of insulin to control excessive nicotine intake in persons with diabetes.

## Introduction

Persons with metabolic disorders, such as diabetes experience compounded health consequences (cardiovascular disease, cancer, and stroke) and higher mortality rates following chronic nicotine use [1–4]. Obese individuals that are diagnosed with Type 2 diabetes (T2D) are more likely to use tobacco products, perhaps due to the ability of nicotine to suppress appetite and control weight gain [5–7]. Persons with diabetes display lower smoking cessation rates, and they express greater concern about gaining weight if they cease their nicotine consumption as compared to smokers without diabetes [8, 9]. The latter reports also show that compared to the general population, persons with diabetes report slightly higher rates of smoking (12.3%) as compared to non-diabetic persons (8.6%). Despite relatively similar rates of smoking behavior, nicotine use in persons with diabetes is concerning given that nicotine exacerbates insulin resistance and facilitates the development of T2D [10, 11]. Given the health challenges associated with insulin resistance and concurrent nicotine use, it is essential to understand the effects of diabetes medications on nicotine use. The present study also included concurrent measures of weight gain as well as food and water intake as additional indices of the effects of our pharmacological agents in insulin resistant rats. These pre-clinical data are essential for the development and safe application of medications that might reduce nicotine intake in persons with diabetes.

With the recent rise in obesity rates, there has been a great deal of interest in finding pharmacological interventions that improve the health outcomes of persons living with T2D. There are new medications that improve the metabolic profile of people with T2D while also reducing their nicotine use, such as GLP-1r agonists [12]. With these data in mind, the present study compared the effects of commonly prescribed medications for diabetes (dapagliflozin, insulin, and bromocriptine) on nicotine intake in female and male rats displaying insulin resistance. Dapagliflozin (Farxiga^®^) is a medication that lowers blood glucose levels in individuals with T2D via inhibition of renal glucose reabsorption through blockade of sodium-glucose cotransporter 2 (SGLT2) which increases urinary glucose excretion [13]. Insulin (Humulin R^®^) is a widely prescribed medication for managing the symptoms of diabetes by promoting glucose uptake into cells and decreasing glucose levels in the body [14, 15]. Bromocriptine (Cycloset^®^) is used to improve insulin sensitivity and enhance glucose homeostasis by stimulating dopamine receptors in the brain which decreases prolactin levels and increases insulin receptor sensitivity [16]. Given the primary role of activation of dopamine systems in motivated behaviors, bromocriptine may reduce the development of nicotine dependence while also alleviating the negative health consequences of T2D [17].

Using rodent models, previous work has assessed the effects of insulin dysregulation on the behavioral effects of nicotine, as reviewed in [18]. Work in our laboratory has revealed that administration of streptozotocin (STZ), a drug that causes hypoinsulinemia by destroying insulin-producing β-cells, enhances conditioned place preference (CPP) and intravenous self-administration (IVSA) [19, 20]. More recently, a report from our laboratory revealed that consumption of a high-fat diet (HFD) increased nicotine IVSA, and this effect was greater in female versus male rats [21]. The latter study included administration of a low dose of STZ to induce insulin resistance after 4 weeks of HFD feeding. This protocol has been used to reliably induce insulin resistance in rats following a chronic HFD feeding regimen [22, 23]. Indeed, this protocol produces cardiac and metabolic dysfunction as well as hyperglycemia and hypertriglyceridemia [24]. The combination of HFD+STZ simulates the prolonged HFD intake, obesity, pancreatic β-cell dysfunction, and the emergence of insulin resistance.

The present study assessed the impact of three different medications used to treat diabetes on nicotine intake in female and male rats. The reinforcing effects of nicotine were assessed using 23 h to nicotine IVSA with 3 days intervening days of abstinence. During the IVSA sessions, changes in body weight, food intake, and water consumption were also examined. Here we are testing the hypothesis that pharmacological interventions that reduce insulin resistance will also decrease nicotine consumption in rats. This hypothesis is based upon prior research demonstrating that insulin supplementation reduced the reinforcing effects of nicotine in STZ-treated rats [25]. Additionally, we predict female rats will self-administer higher amounts of nicotine when compared to males.

## Materials and methods

### Subjects

Outbred female and male Wistar rats (*n* = 35 total) from Envigo Inc., United States were housed in a humidity- and temperature-controlled vivarium using a 12 h light/dark cycle with lights off at 6:00 p.m. Rats were *ad-libitum* fed either regular diet (RD; 3.1 kcal/g, 17% kcal from fat) or a HFD (5.1 kcal/g, 60% kcal from fat). The food was purchased from Envigo Teklad (Madison, WI, United States #TD.06414). All rats were handled for 3–5 days prior to the start of the experiment. The animals were cared for in compliance with the Guide for the Care and Use of Laboratory Animals, and all procedures were approved by the UTEP Institutional Animal Care and Use Committee.

### Experimental design

**FIGURE 1:**
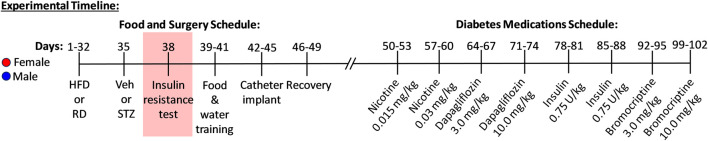
Timeline and design of experimental procedures. Female (red symbol) and male (blue symbol) rats received a regular diet (RD) or high fat diet (HFD) for 32 days. On day 35, rats received vehicle or streptozotocin (STZ; 25 mg/kg) followed by an insulin challenge to assess insulin resistance (day 38). All rats were subjected to food and water training for 3 days in the operant chambers (days 39-41) followed by catheter surgeries and recovery (days 42-49). All rats were then introduced to nicotine intravenous self-administration (IVSA) that occurred for 2 dose cycles (0.015 and 0.03 mg/kg) for 4 days each prior to receiving administration of diabetes pharmacotherapies (days 50-60). Rats then received various medications for diabetes, including dapagliflozin (days 64-74; 3.0 and 10.0 mg/kg), insulin (days 78-88; 0.75 U/kg), and bromocriptine (days 92-102; 3.0 and 10.0 mg/kg). Nicotine, food, water, and weight changes were recorded during IVSA testing.

The present study assessed the effects of 3 different FDA-approved agents used to treat diabetes: dapagliflozin (Farxiga^®^), insulin (Humulin R^®^), and bromocriptine (Cycloset^®^). These drugs were included because they are commonly prescribed in persons with T2D and they differ with respect to their direct or indirect regulation of glucose levels. Separate groups of rats received a RD (*n* = 4 female and 5 male) or a HFD (n = 7 female and 8 male) for 4 weeks. These rats then received a low dose of STZ (25 mg/kg, subcutaneous), and 3 days later the rats were given an insulin resistance test, as described below. A group of RD rats that did not receive the low dose of STZ (*n* = 6 female and 5 male) were also included to assess the potential effects of STZ alone. The results revealed that nicotine intake was similar in RD (40.16 ± 10.64) versus RD+STZ (34.38 ± 6.31) during IVSA of the 0.03 mg/kg dose of nicotine. Similarly, food and water intake as well as changes in body weight were similar in RD versus RD+STZ controls. These data suggest that a low dose of STZ does not impact nicotine intake on its own and that the RD+STZ group provides an appropriate control comparison for the pharmacological effects observed in HFD-fed rats. During each session, nicotine lever presses, weight changes, food intake, and water responses were concomitantly recorded. Figure 1 depicts our experimental timeline.

### Drugs

(−) Nicotine hydrogen tartrate salt was obtained from the NIDA Drug Supply Program (Research Triangle, Bethesda, MD) and was dissolved in 0.9% sterile saline (pH 7.4). The nicotine solutions were prepared fresh every day and were adjusted to the rats’ body weight from the previous day. The STZ was purchased from Millipore Sigma (St. Louis, MO) and was dissolved in a citrate buffer (0.1 M citric acid and 0.1 M Na citrate, pH 6.0). Fresh solutions of STZ were administered within 15 min of preparation for each animal. Insulin was obtained from Humulin^®^ (Indianapolis, IN) and was dissolved in 0.9% sterile saline (pH 7.4). Dapagliflozin was dissolved in propylene glycol (100%) and bromocriptine mesylate was dissolved in a water/propylene glycol (100%) solution (60/40). Bromocriptine and dapagliflozin were purchased from Millipore Sigma (St. Louis, MO). Cefazolin^®^ antibiotic was purchased from Henry Schein (Melville, NY) and was dissolved in a heparin solution. All rats received all doses and drugs.

### Feeding regimen and insulin challenge test

The rats were fed a RD (3.1 kcal/g, 17% kcal from fat) or a HFD (5.1 kcal/g, 60% kcal from fat) that was purchased from Envigo Teklad (Madison, WI, Catalogue number: TD.06414). Rats were given *ad libitum* access to their respective diet throughout the experimental timeline. The HFD was stored at 4°C and both diets were replenished each day at 11 a.m. when the food intake and body weights were recorded. On Day 35 of the feeding regimen, the rats received an injection of STZ (25 mg/kg, subcutaneous). Three days later, the rats were tested for insulin resistance following a 16 h food deprivation period. The rats were given an injection of insulin (0.75 U/kg; intraperitoneal), and blood samples were collected 15, 30, 60, 120, and 180 min later. The blood samples were collected using a lancet to prick the tip of the tail to extract a small drop of blood that was placed on a glucose test strip. Glucose levels were analyzed using a glucose meter that was calibrated for rodent blood (AlphaTRAK^®^, Abbott Park, IL). Their respective food regimen was replaced immediately after the insulin resistance test.

### IVSA testing

The IVSA procedures were based on previous work in our laboratory using 23 h access to nicotine IVSA in rats [19, 20]. The day after the insulin resistance test, the rats were placed into operant chambers where they had access to a feeding cup that contained their respective diet. The rats were also allowed to nosepoke in a hole that activated the delivery of 0.1 mL aliquots of water into an adjacent metal dipper cup on a fixed ratio-1 (FR-1) schedule of reinforcement. All rats reached stable levels of water responding prior to catheter surgery, which occurred between Days 42–45 of the feeding regimen. The rats were anesthetized using an isoflurane/oxygen vapor mixture (1%–3%) and were then prepared with IV catheters into the jugular vein, as previously described [21]. The rats were given a 4–7 day recovery period in their home cage with *ad libitum* access to water and their respective RD or HFD. The catheters were flushed daily (0.2 mL) with an antibiotic solution containing Cefazolin^®^ and heparin (30 USP units/mL). Following recovery, the rats were placed back into the operant chambers where they were given 23 h access to nicotine IVSA on an FR-1 schedule of reinforcement for different doses of nicotine (0.015 and then 0.03 mg/kg/0.1 mL infusion). Each dose was available for 4 consecutive days with 3 intervening days of forced abstinence in their home cage. The active lever delivered a 1 s infusion of nicotine followed by a 20 s time-out period where responses were recorded but had no consequences. Responses on the inactive lever were recorded but had no scheduled consequences. In the next phases of the study, the rats had access to the same dose of nicotine (0.03 mg/kg, intravenous), but the rats received administration of two doses of three different medications that are used clinically to decrease insulin resistance. Each day of IVSA testing, the rats were removed from their operant chamber at the onset of their dark cycle (6 p.m.) and were injected with the same dose of the medication that was being tested that week. Specifically, the rats first received dapagliflozin at a dose of 3.0 mg/kg (subcutaneous) for four nights, and the following week they received 10.0 mg/kg (subcutaneous) for another four nights. These doses were based on previous work comparing the effects of nicotine CPP in HFD-fed rats [26]. The following week, the rats received insulin at a dose of 0.75 U/kg (intraperitoneal), and the next week they received the same dose for another 4 nights. The dose of insulin was used based on a prior study comparing nicotine intake in HFD-fed rats [21]. In the last phase of the study, the rats received bromocriptine at a dose of 3.0 mg/kg (subcutaneous) for 4 nights, and the following week they received 10.0 mg/kg for another four nights. The high dose of bromocriptine has been shown to alter gastrointestinal transit via a dopamine-mediated mechanism [27]. During each IVSA session, lever presses for nicotine, weight change, food intake, and water responses were recorded.

### Statistical analyses

Glucose levels were analyzed using a 3-way analysis of variance (ANOVA) with Diet (RD versus HFD) and Sex (female versus male) as between subject factors and Time as a within-subject factor. The food intake data were adjusted to reflect caloric value of the RD (*g x 3.1*) or the HFD (*g x 5.1*). Also, the rats’ weights were expressed as % weight change (*daily value−initial value*) ÷ *initial value × 100%*) relative to the first day of IVSA for each treatment. An initial analysis that included Diet and Sex as between-subject factors and Dose as a within-subject factor revealed that there were no significant differences across Dose of both nicotine and treatments. Since our effects were similar across doses of nicotine and the pharmacological agents, the subsequent analyses included 2-way ANOVAs that included Diet and Sex as between-subject factors. All datasets depict individual female (shown in red) and male (shown in blue) rats. Where sex differences were not observed, the analyses collapsed across female and male rats. However, where significant interactions were observed, *post hoc* analyses were conducted, and Bonferroni corrections were applied to the resultant *p* values. All graphs were generated using Graphpad Prism version 8 and analyzed on SPSS IBM software.

## Results

**FIGURE 2:**
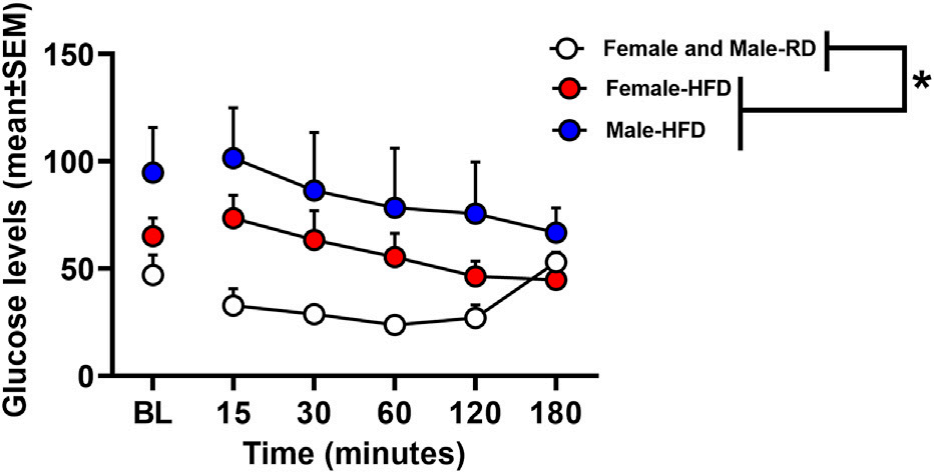
Glucose levels expressed in mg/dL following an insulin challenge in rats that received a RD or a HFD. The data are expressed as mean (±SEM). The red symbols reflect females and blue symbols reflect males that were fed a HFD. The white symbols reflect both female and male RD controls. Blood samples were collected during baseline and 15, 30, 60, 120, and 180 min after an injection of insulin (0.75 U/kg). The asterisks (*) denote a difference from RD controls (*p* ≤ 0.05).

Figure 2 depicts glucose levels in female and male rats that were fed a RD or a HFD. The analysis revealed that there was no interaction between Diet, Sex, and Time [*F*_(1,20)=_0.66, *p* = 0.43]. However, there was a main effect of Diet [*F*_(1,20)=_8.00, *p* = 0.01], with rats that were fed a HFD displaying higher glucose levels as compared to RD controls regardless of sex. Accordingly, there was no main effect of Sex [*F*_(1,20)=_2.11, *p* = 0.16].

**FIGURE 3:**
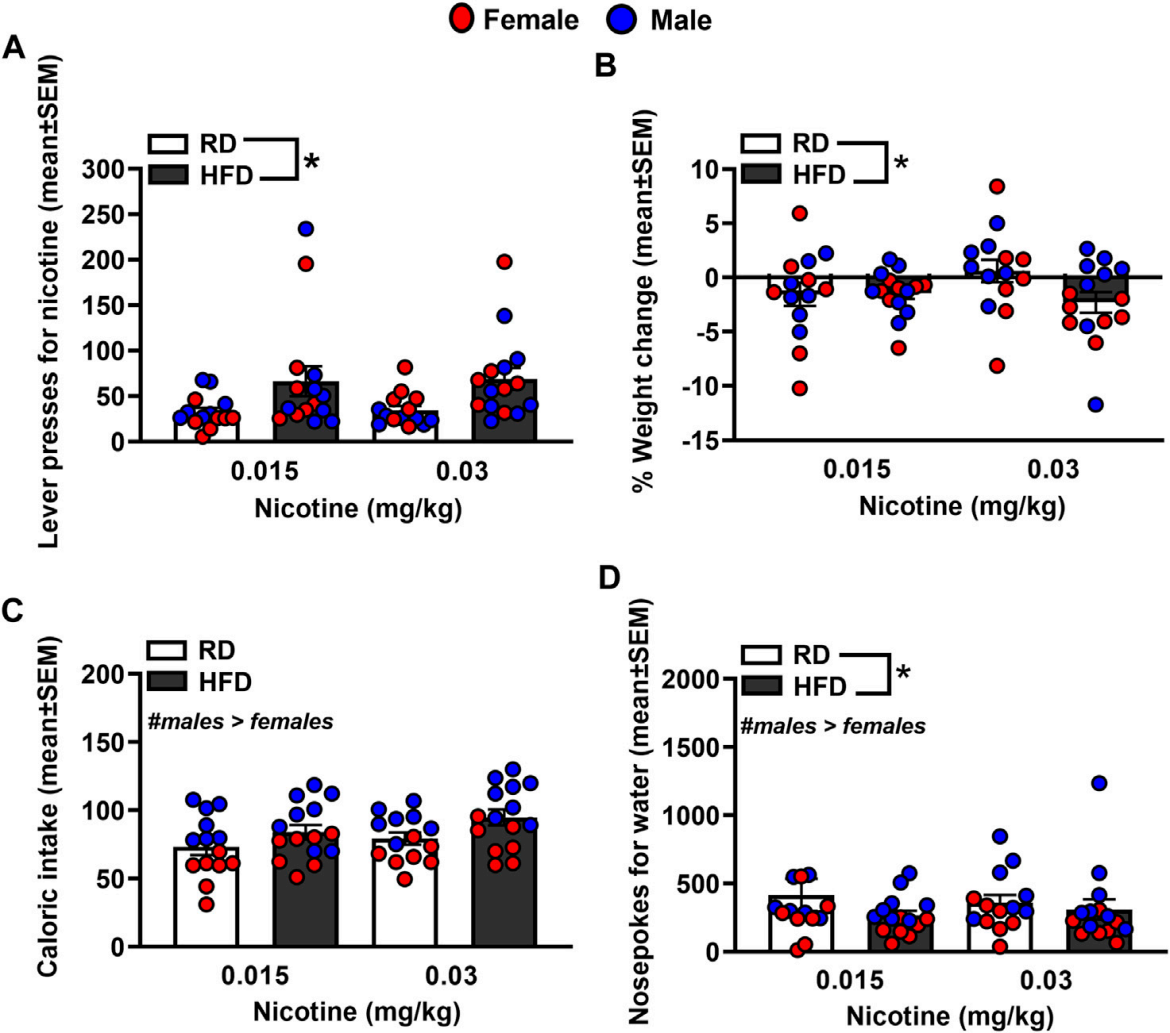
Lever presses for nicotine **(A)**, %weight change **(B)**, caloric intake **(C)**, and nosepokes for water **(D)** during IVSA of nicotine. The data are expressed as mean values across the 4 days of IVSA (±SEM). The red symbols reflect females and the blue symbols reflect males that were fed a RD (white bars) or a HFD (black bars). The number signs denote (#) a significant sex difference, the asterisks (*) denote a difference from RD controls (*p* ≤ 0.05).

Figure 3 depicts lever presses for nicotine, % weight change, caloric intake, and nosepoke responses for water in rats that were fed a RD or a HFD. The analysis of nicotine lever presses revealed there was no interaction between Diet and Sex [*F*_(1,44)=_ 0.23, *p* = 0.63]. There was a main effect of Diet [*F*_(1,44)=_8.14, *p* = 0.01], but not Sex [*F*_(1,44)_ = 0.001, *p* = 0.98]. Specifically, rats that were fed a HFD displayed more lever presses for nicotine as compared to RD controls regardless of sex. The analysis of % weight change revealed there was no interaction between Diet and Sex [*F*_(1,44)=_0.003, *p* = 0.96]. There was a main effect of Diet [*F*_(1,44)_ = 6.89, *p* = 0.01], but not Sex [*F*_(1,44)_ = 1.24, *p* = 0.27], with HFD rats displaying greater weight change than RD controls regardless of sex. The analysis of caloric intake revealed there was no interaction between Diet and Sex [*F*_(1,44)_ = 0.48, *p* = 0.49]. There was a main effect of Sex [*F*_(1,44)_ = 11.68, *p* = 0.001], but not Diet [*F*_(1,44)_ = 0.05, *p* = 0.82], with males consuming more calories than females regardless of their diet regimen. The analysis of nosepokes for water revealed there was no interaction between Diet and Sex [*F*_(1,44)_ = 0.09, *p* = 0.77]. However, there were significant main effects of Diet [*F*_(1,44)_ = 3.89, *p* = 0.05] and Sex [*F*_(1,44)_ = 44.41, *p* ≤ 0.001], with rats that were fed a HFD responding more for water than RD controls and males consuming more water than females.

**FIGURE 4:**
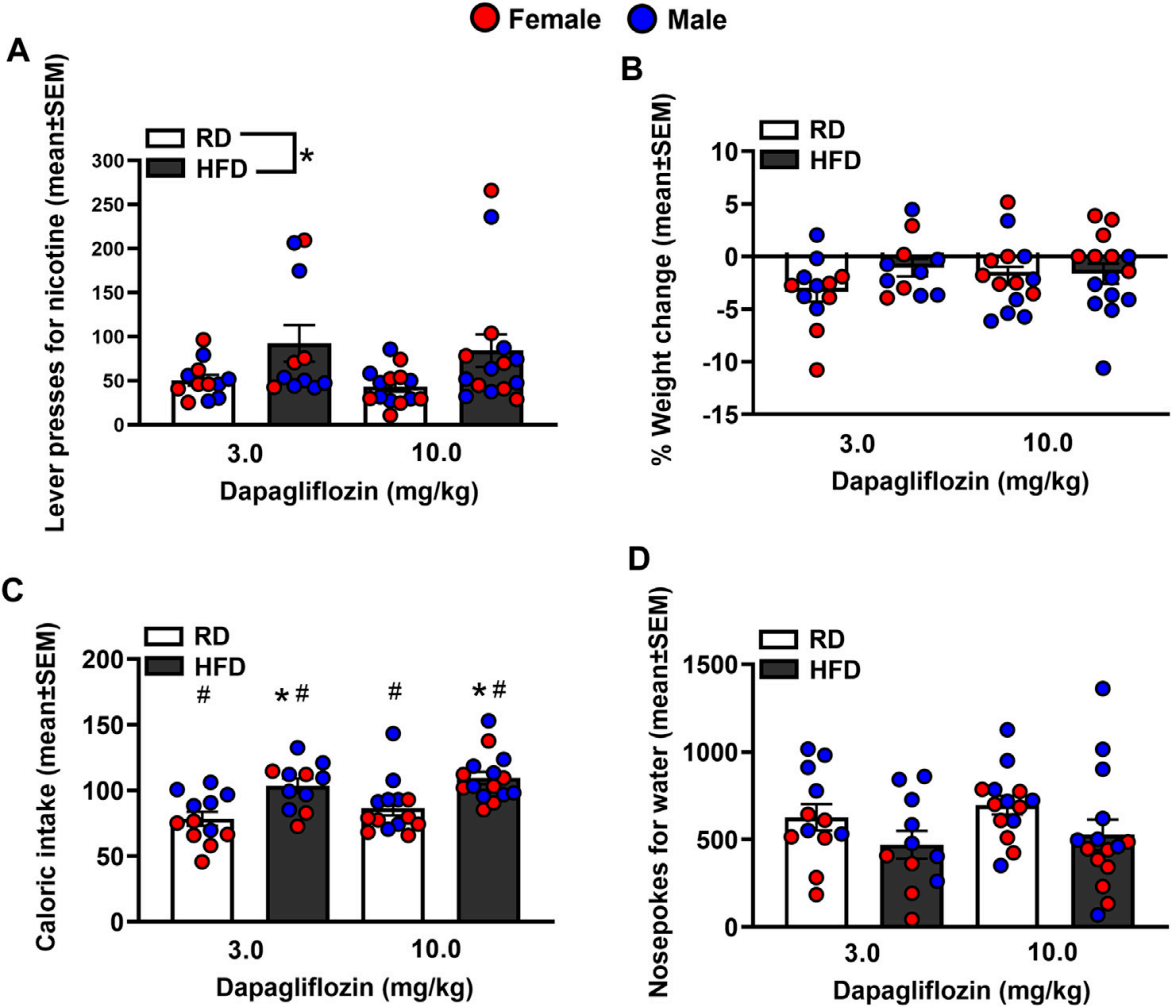
Lever presses for nicotine **(A)**, %weight change **(B)**, caloric intake **(C)**, and nosepokes for water **(D)** following administration of dapagliflozin. The data are expressed as mean values across the 4 days of IVSA (±SEM). The red symbols reflect females and the blue symbols reflect males that were fed a RD (white bars) or a HFD (black bars). The number signs denote (#) a significant sex difference, the asterisks (*) denote a difference from RD controls (*p* ≤ 0.05).

Figure 4 depicts lever presses for nicotine, % weight change, caloric intake, and nosepoke responses for water in rats were treated with dapagliflozin. The analysis of nicotine intake revealed there was no interaction between Diet and Sex [*F*_(1,38)=_ 0.065, *p* = 0.80]. There was a main effect of Diet [*F*_(1,38)_ = 8.23, *p* = 0.01], but not Sex [*F*_(1,38)_ = 0.21, *p* = 0.65]. Rats that were fed a HFD displayed more lever presses for nicotine as compared to RD controls regardless of sex. The analysis of % weight change revealed there was no interaction between Diet and Sex [*F*_(1,38)_ = 1.27, *p* = 0.27]. Also, there was no main effect of Diet [*F*_(1,38)_ = 0.032, *p* = 0.86] or Sex [*F*_(1,38)_ = 3.82, *p* = 0.58]. The analysis of caloric intake revealed there was an interaction between Diet and Sex [*F*_(1,38)_ = 6.84, *p* = 0.01]. Post hoc analyses revealed that male HFD rats consumed more calories than male RD controls (*p* < 0.05). The analysis of nosepokes for water revealed there was no interaction between Diet and Sex [*F*_(1,38)_ = 3.36, *p* = 0.08]. Also, there were no main effects of Diet [*F*_(1,38)_ = 3.21, *p* = 0.08] or Sex [*F*_(1,38)_ = 1.23, *p* = 0.27].

**FIGURE 5:**
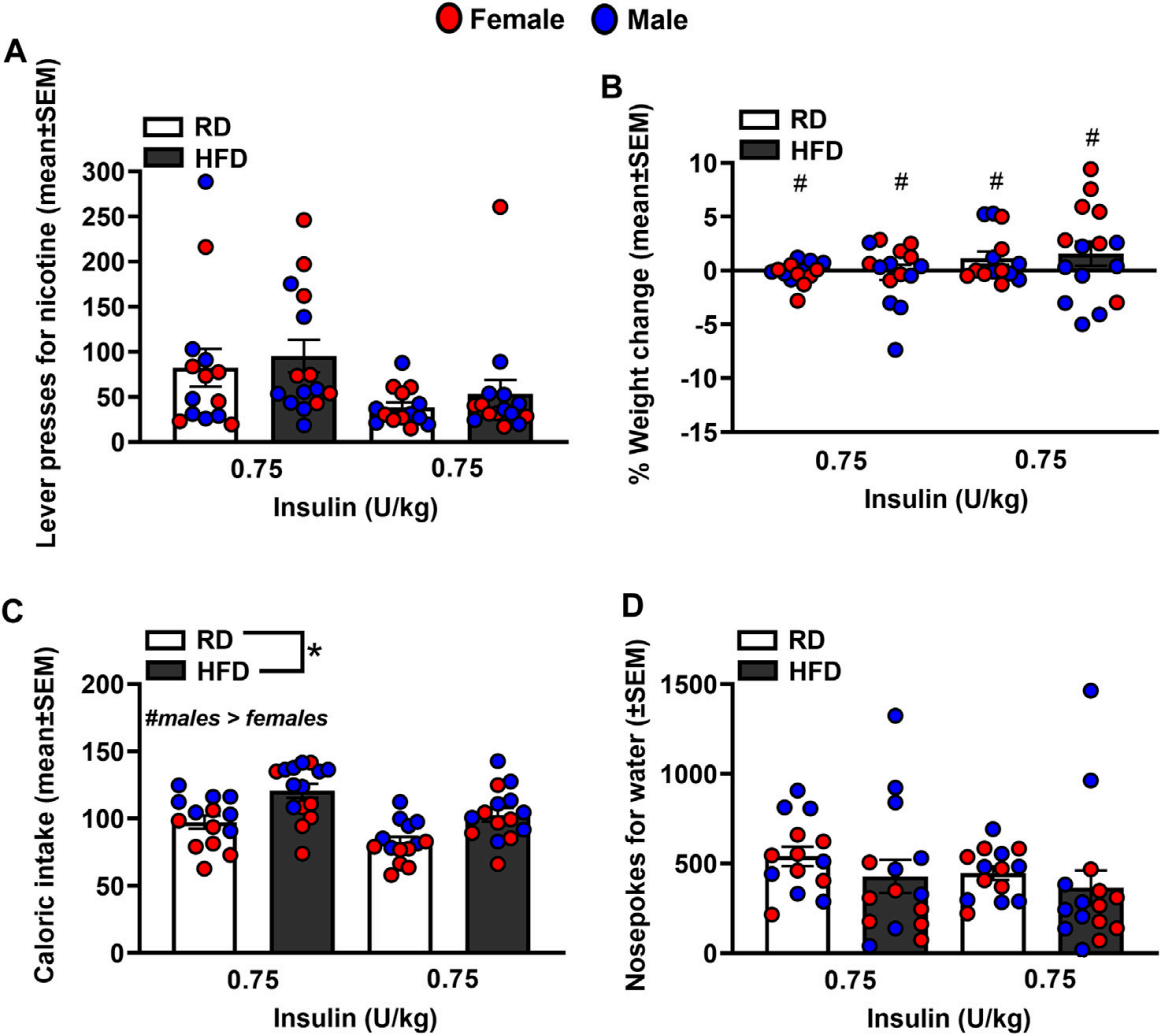
Lever presses for nicotine **(A)**, %weight change **(B)**, caloric intake **(C)**, and nosepokes for water **(D)** following administration of insulin. The data are expressed as mean values across the 4 days of IVSA (±SEM). The red symbols reflect females and the blue symbols reflect males that were fed a RD (white bars) or a HFD (black bars). The number signs denote (#) a significant sex difference, the asterisks (*) denote a difference from RD controls (*p* ≤ 0.05).

Figure 5 depicts lever presses for nicotine, % weight change, caloric intake, and nosepoke responses for water in rats that were treated with insulin. The analysis of nicotine intake revealed there was no interaction between Diet and Sex [*F*_(1,44)_ = 2.20, *p* = 0.15]. Also, there were no main effects of Diet [*F*_(1,44)_ = 0.66, *p* = 0.42] or Sex [*F*_(1,44)_ = 0.25, *p* = 0.62]. The analysis of % weight change revealed there was an interaction between Diet and Sex [*F*_(1,44)_ = 3.92, *p* = 0.05]. Post hoc analyses revealed that female rats displayed a greater increase in body weight than males regardless of their diet regimen (*p* < 0.05). The analysis of caloric intake revealed there was no interaction between Diet and Sex [*F*_(1,44)_ = 0.28, *p* = 0.60]. However, there were significant main effects of Diet [*F*_(1,44)_ = 15.17, *p* ≤ 0.001] and Sex [*F*_(1,44)_ = 11.54, *p* = 0.001]. Rats that were given access to a HFD consumed more calories than RD controls and males consumed more calories than females. The analysis of nosepokes for water revealed there was no interaction between Diet and Sex [*F*_(1,44)_ = 1.32, *p* = 0.26]. There were also no main effects of Diet [*F*_(1,44)_ = 0.90, *p* = 0.35] or Sex [*F*_(1,44)_ = 3.02, *p* = 0.09].

**FIGURE 6:**
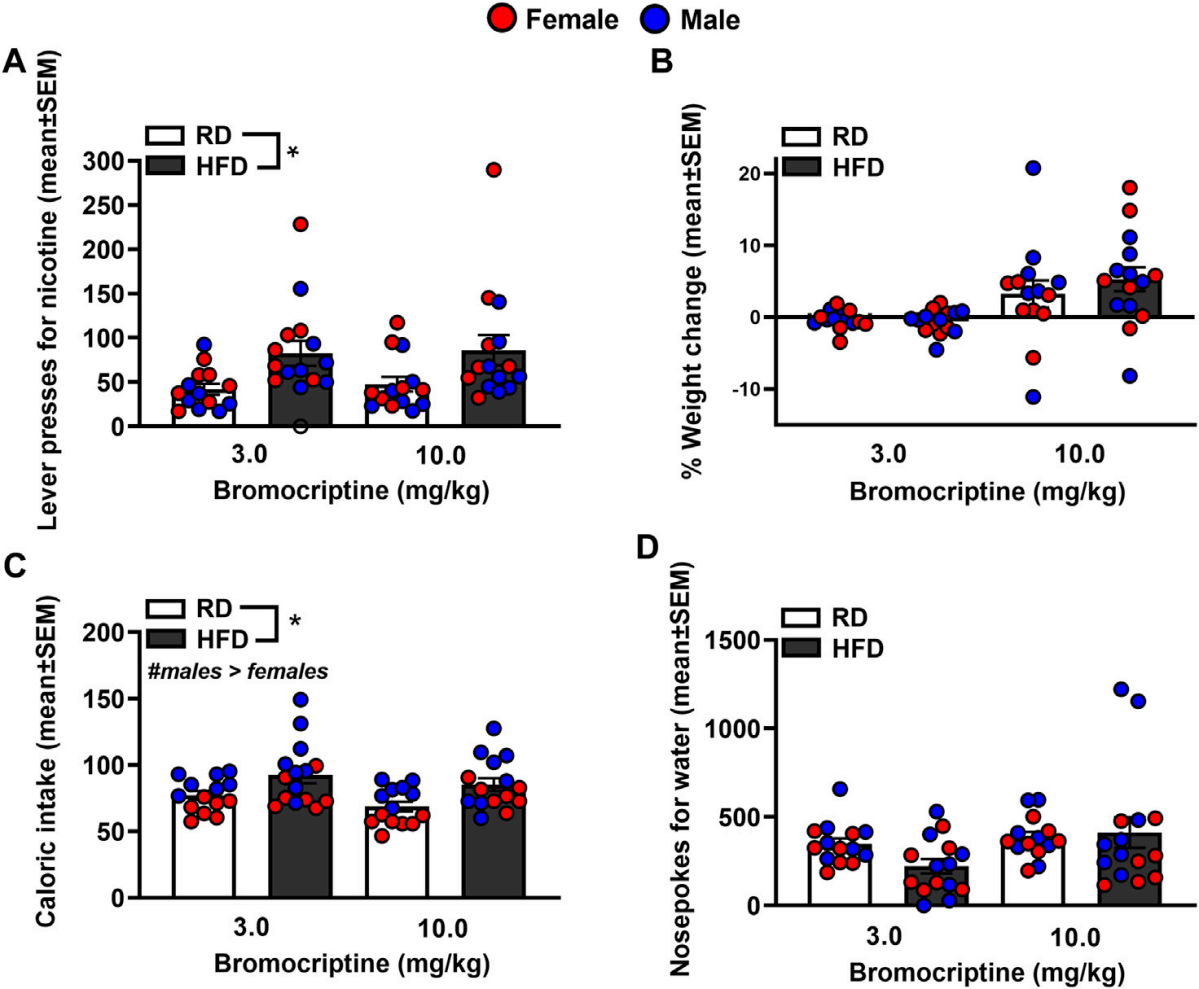
Lever presses for nicotine **(A)**, %weight change **(B)**, caloric intake **(C)**, and nosepokes for water **(D)** following administration of bromocriptine. The data are expressed as mean values across the 4 days of IVSA (±SEM). The red symbols reflect females and the blue symbols reflect males that were fed a RD (white bars) or aHFD (black bars). The number signs denote (#) a significant sex difference, the asterisks (*) denote a difference from RD controls (p ≤ 0.05).

Figure 6 depicts lever presses for nicotine, % weight change, caloric intake, and nosepoke responses for water in rats that were treated with bromocriptine. The analysis of nicotine intake revealed there was no interaction between Diet and Sex [*F*_(1,44)_ = 2.53, *p* = 0.12]. There was a significant main effect of Diet [*F*_(1,44)_ = 11.88, *p* = 0.001], but not Sex [*F*_(1,44)_ = 0.40, *p* = 0.53]. Rats that were given access to a HFD displayed more lever presses for nicotine as compared to RD controls regardless of sex. The analysis of % body weight revealed there was no interaction between Diet and Sex [*F*_(1,44)_ = 0.39, *p* = 0.54]. There were also no main effects of Diet [*F*_(1,44)_ = 0.002, *p* = 0.97] or Sex [*F*_(1,44)_ = 0.000, *p* = 1.00]. The analysis of caloric intake revealed there was no interaction between Diet and Sex [*F*_(1,44)_ = 0.04, *p* = 0.84]. However, there were significant main effects of Diet [*F*_(1,44)_ = 11.12, *p* = 0.002] and Sex [*F*_(1,44)_ = 21.44, *p* ≤ 0.001], with HFD rats consuming more calories than RD controls and males consuming more calories than females. The analysis of nosepokes for water revealed there was no interaction between Diet and Sex [*F*_(1,44)_ = 0.94, *p* = 0.34]. There were also no main effects of Diet [*F*_(1,44)_ = 0.34, *p* = 0.57] or Sex [*F*_(1,44)_ = 2.16, *p* = 0.15].

## Discussion

To summarize, the challenge injection of insulin decreased glucose levels in RD controls regardless of sex. The ability of insulin to lower glucose levels was suppressed in HFD-fed female and male rats. These data serve as an indicator of the development of insulin resistance in our experimental protocol. A summary of our effects is provided in Table 1. Prior to any treatment interventions, all the HFD-fed rats displayed higher levels of nicotine intake as compared to RD controls. As one might expect, the HFD-fed rats also gained less weight than RD controls likely due to the appetite suppressant effects of nicotine [28]. Regardless of diet, all male rats displayed higher caloric intake than females, consistent with prior work [29]. All the HFD-fed rats displayed more water intake than RD controls, which is another indicator of the development of insulin resistance in our protocol. Of the diabetes medications that were examined, insulin was the only drug that normalized the high levels of nicotine intake in HFD-fed rats to RD control levels. Dapagliflozin and bromocriptine administration normalized the weight gain that was observed in HFD-fed rats. This finding is consistent with prior work showing that bromocriptine and dapagliflozin reduce body weight, improve glucose tolerance, catabolic function, and tissue adiposity [30, 31]. The high levels of caloric intake observed in HFD-fed male rats was not altered by any of our pharmacological interventions. In contrast, all our pharmacological interventions normalized the high levels of water intake observed in HFD-fed rats.

**TABLE 1:** Summary of effects in HFD versus RD controls.

Treatment:	Nicotine Intake	Weight change	Caloric intake	Water intake
No treatment	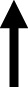	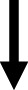	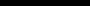	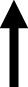
Dapagliflozin	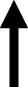	normalized	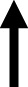	normalized
Insulin	normalized	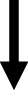	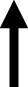	normalized
Bromocriptine	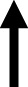	normalized	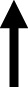	normalized

This table reflects summary of effects on nicotine, food, water, and weight in HFD-versus RD-treated rats. Grey shading denotes where sex differences were observed. The dash denotes that there was similar caloric intake across diet groups. The term normalized was used to denote where the effects of the HFD were similar to RD controls.

The major goal of this study was to assess the impact of diabetes medications on nicotine intake in a rodent model that induces insulin resistance. Prior studies in rodents have assessed insulin resistance by giving a challenge injection of insulin and measuring the degree to which peripheral glucose levels are reduced [32]. The emergence of insulin resistance is evidenced by the inability of insulin to lower glucose from baseline levels [25]. The present study employed a 4 weeks HFD feeding regimen followed by an injection of a low dose of STZ. In response to a challenge injection of insulin, the HFD-fed rats displayed higher glucose levels than RD controls, suggesting that our feeding regimen combined with administration of a low dose of STZ induced insulin resistance. There was a non-significant trend for males to display slightly higher levels of glucose than females, an effect that may have been expected from prior studies comparing sex differences in plasma glucose levels [21, 26].

A major focus of this study was to examine whether diabetes medications alter the reinforcing effects of nicotine. We first compared the effects of HFD versus RD on IVSA of different doses of nicotine. The results revealed that HFD-fed rats displayed significantly higher nicotine intake than RD controls, consistent with a prior nicotine IVSA study in insulin resistant rats [21]. The finding that a disruption in insulin systems increases nicotine reward is consistent with a prior report showing that a subthreshold dose of nicotine only produced CPP in a sub-set of HFD-fed rats that also displayed insulin resistance [26]. In support of the role of insulin in modulating nicotine intake, the present study also revealed that dapagliflozin and bromocriptine, which reduce glucose levels via an insulin-*independent* mechanism, did not alter the excessive nicotine intake observed in our insulin resistant rats.

Regarding sex differences, the present study revealed that male rats displayed higher caloric intake than females during the nicotine IVSA sessions and following administration of the diabetes medications. This pattern of results is consistent with prior work showing that male rats consume more calories than females [29]. Importantly, our results revealed that insulin was equally effective at normalizing the excessive nicotine intake in HFD-fed female and male rats. These data suggest that clinical interventions employing insulin supplementation may be equally effective in managing diabetes and promoting nicotine cessation in females and males in a clinical setting.

There are some limitations to consider with the present study. First, our within-subject design utilized repeated administration of the pharmacotherapies in the same rats across time. Thus, each drug treatment may have impacted the effects of the subsequent pharmacotherapies. Our pharmacotherapies may have also improved the metabolic profile and the blood glucose levels of our HFD-fed rats across our treatment regimen. Future studies are needed to determine whether changes in nicotine intake coincide with improvements in plasma and/or brain biomarkers of metabolic syndrome. Future work is also needed to determine whether our pharmacotherapies normalize blood glucose levels as the rats are self-administering nicotine. The recent introduction and widespread use of glucagon-like peptide 1 receptor (GLP-1R) agonists presents new opportunities to study the therapeutic potential of these anti-obesity medications on nicotine dependence in individuals displaying T2D. Indeed, recent reports have revealed that GLP-1 agonists reduce substance use and alcohol dependence [12, 33]. Thus, future studies might examine the effects of novel medications that stimulate GLP-1Rs on excessive nicotine intake in rodent models of diabetes. Future studies might also examine the effects of other diabetes medications such as metformin on nicotine intake in insulin resistant rats.

In conclusion, the present study suggests that direct activation of insulin systems is necessary for reducing the excessive nicotine intake observed in insulin-resistant rats. These data imply that medications that directly activate insulin may be most effective in reducing nicotine use in persons with diabetes. Regarding mechanisms, our prior work revealed that hypoinsulinemia suppresses dopamine transmission in the mesolimbic reward pathway, and this effect was normalized to control levels following insulin supplementation [34]. Thus, excessive nicotine intake produced by insulin resistance is likely modulated via insulin modulation of dopamine transmission. A prior clinical report revealed that the dopamine agonist, bromocriptine, reduced cigarette smoking in healthy smokers [25]. Thus, future studies are needed to understand the complex interplay between insulin and dopamine in the progression of nicotine dependence in persons with diabetes.

## Data Availability

The raw data supporting the conclusion of this article will be made available by the authors, without undue reservation.

## References

[1] Al-Delaimy WK Manson JE Solomon CG Kawachi I Stampfer MJ Willett WC Smoking and risk of coronary heart disease among women with type 2 diabetes mellitus. Arch Intern Med (2002) 162(3):273–9. 10.1001/archinte.162.3.273 11822919

[2] Lycett D Nichols L Ryan R Farley A Roalfe A Mohammed MA The association between smoking cessation and glycaemic control in patients with type 2 diabetes: a THIN database cohort study. Lancet Diabetes Endocrinol (2015) 3:423–30. 10.1016/S2213-8587(15)00082-0 25935880

[3] Sliwinska-Mosson M Milnerowicz H. The impact of smoking on the development of diabetes and its complications. Diabetes Vasc Dis Res (2017) 14(4):265–76. 10.1177/1479164117701876 28393534

[4] Winhusen T Theobald J Kaelber DC Lewis D. Medical complications associated with substance use disorders in patients with type 2 diabetes and hypertension: electronic health record findings. Addiction (2019) 114(8):1462–70. 10.1111/add.14607 30851217 PMC6626564

[5] Chen H Saad S Sandow SL Bertrand PP. Cigarette smoking and brain regulation of energy homeostasis. Front Pharmacol (2012) 3:147. 10.3389/fphar.2012.00147 22848202 PMC3404499

[6] White MA McKee SA O’Malley SS. Smoke and mirrors: magnified beliefs that cigarette smoking suppresses weight. Addict Behav (2007) 32(10):2200–10. 10.1016/j.addbeh.2007.02.011 17428615 PMC1993360

[7] White MA. Smoking for weight control and its associations with eating disorder symptomatology. Compr Psychiatr (2012) 53(4):403–7. 10.1016/j.comppsych.2011.05.007 PMC3193869 21741037

[8] Bishop FK Maahs DM Snell-Bergeon JK Ogden LG Kinney GL Rewers M. Lifestyle risk factors for atherosclerosis in adults with type 1 diabetes. Diabetes Vasc Dis Res (2009) 6(4):269–75. 10.1177/1479164109346359 20368221

[9] Gill GV Morgan C MacFarlane IA. Awareness and use of smoking cessation treatments among diabetic patients. Diabetic Med a J Br Diabetic Assoc (2005) 22(5):658–60. 10.1111/j.1464-5491.2005.01471.x 15842526

[10] Brath H Kaser S Tatschl C Fasching P. Smoking, alcohol and diabetes (Update 2019). Wien Klin Wochenschr (2019) 131(1):67–70. 10.1007/s00508-019-1455-z 30980165

[11] Maddatu J Anderson-Baucum E Evans-Molina C. Smoking and the risk of type 2 diabetes. Translational Res J Lab Clin Med (2017) 184:101–7. 10.1016/j.trsl.2017.02.004 PMC5429867 28336465

[12] Tuesta LM Chen Z Duncan A Fowler CD Ishikawa M Lee BR GLP-1 acts on habenular avoidance circuits to control nicotine intake. Nat Neurosci (2017) 20(5):708–16. 10.1038/nn.4540 28368384 PMC5541856

[13] Ferrannini E Ramos SJ Salsali A Tang W List JF. Dapagliflozin monotherapy in type 2 diabetic patients with inadequate glycemic control by diet and exercise: a randomized, double-blind, placebo-controlled, phase 3 trial. Diabetes care (2010) 33(10):2217–24. 10.2337/dc10-0612 20566676 PMC2945163

[14] Anderson JH Brunelle RL Koivisto VA Pfützner A Trautmann ME Vignati L Reduction of postprandial hyperglycemia and frequency of hypoglycemia in IDDM patients on insulin-analog treatment. Multicenter Insulin Lispro Study Group. Diabetes (1997) 46(2):265–70. 10.2337/diab.46.2.265 9000704

[15] Chen J Borra S Huang A Fan L Pollom RD Hood RC. Treatment patterns and outcomes before and after humulin R U-500 initiation among US patients with type 2 diabetes previously prescribed ≤ 200 units/day of U-100 insulin. Diabetes Ther (2022) 13(3):465–79. 10.1007/s13300-022-01209-z 35190970 PMC8934887

[16] Lopez Vicchi F Luque GM Brie B Nogueira JP Garcia Tornadu I Becu-Villalobos D. Dopaminergic drugs in type 2 diabetes and glucose homeostasis. Pharmacol Res (2016) 109:74–80. 10.1016/j.phrs.2015.12.029 26748034

[17] Shivaprasad C Kalra S. Bromocriptine in type 2 diabetes mellitus. Indian J Endocrinol Metab (2011) 15(1):S17–24. 10.4103/2230-8210.83058 21847449 PMC3152192

[18] O'Dell LE Nazarian A. Enhanced vulnerability to tobacco use in persons with diabetes: a behavioral and neurobiological framework. Prog neuro-psychopharmacology Biol Psychiatry (2016) 65:288–96. 10.1016/j.pnpbp.2015.06.005 26092247

[19] Pipkin JA Cruz B Flores RJ Hinojosa CA Carcoba LM Ibarra M Both nicotine reward and withdrawal are enhanced in a rodent model of diabetes. Psychopharmacology (2017) 234(9-10):1615–22. 10.1007/s00213-017-4592-y 28342091 PMC5437741

[20] O'Dell LE Natividad LA Pipkin JA Roman F Torres I Jurado J Enhanced nicotine self-administration and suppressed dopaminergic systems in a rat model of diabetes. Addict Biol (2014) 19(6):1006–19. 10.1111/adb.12074 23834715 PMC3842417

[21] Cruz B Ortegon S Giner P Matos-Ocasio F Rodriguez-Crespo A Uribe KP The emergence of insulin resistance following a chronic high-fat diet regimen coincides with an increase in the reinforcing effects of nicotine in a sex-dependent manner. Neuropharmacology (2021) 200:108787. 10.1016/j.neuropharm.2021.108787 34571112 PMC10228837

[22] Mehta BK Banerjee S. Minocycline reverses diabetes-associated cognitive impairment in rats. Pharmacol Rep PR (2019) 71(4):713–20. 10.1016/j.pharep.2019.03.012 31207433

[23] Xiang X Wang Z Zhu Y Bian L Yang Y. Dosage of streptozocin in inducing rat model of type 2 diabetes mellitus. Wei Sheng Yan jiu = J Hyg Res (2010) 39(2):138–42. 20459021

[24] Mansor LS Gonzalez ER Cole MA Tyler DJ Beeson JH Clarke K Cardiac metabolism in a new rat model of type 2 diabetes using high-fat diet with low dose streptozotocin. Cardiovasc diabetology (2013) 12:136–10. 10.1186/1475-2840-12-136 PMC3849358 24063408

[25] Jarvik ME Caskey NH Wirshing WC Madsen DC Iwamoto-Schaap PN Elins JL Bromocriptine reduces cigarette smoking. Addiction (2000) 95(8):1173–83. 10.1046/j.1360-0443.2000.95811734.x 11092065

[26] Íbias J O'Dell LE Nazarian A. Insulin dependent and independent normalization of blood glucose levels reduces the enhanced rewarding effects of nicotine in a rodent model of diabetes. Behav Brain Res (2018) 351:75–82. 10.1016/j.bbr.2018.05.018 29803655 PMC6026546

[27] Dhasmana KM Villalón CM Zhu YN Parmar SS. The role of dopamine (D2), alpha and beta-adrenoceptor receptors in the decrease in gastrointestinal transit induced by dopamine and dopamine-related drugs in the rat. Pharmacol Res (1993) 27(4):335–47. 10.1006/phrs.1993.1033 8103596

[28] Rupprecht LE Smith TT Donny EC Sved AF. Self-administered nicotine differentially impacts body weight gain in obesity-prone and obesity-resistant rats. Physiol Behav (2017) 176:71–5. 10.1016/j.physbeh.2017.02.007 28189503 PMC6044443

[29] la Fleur SE Luijendijk MC van der Zwaal EM Brans MA Adan RA. The snacking rat as model of human obesity: effects of a free-choice high-fat high-sugar diet on meal patterns. Int J Obes (2014) 38(5):643–9. 10.1038/ijo.2013.159 23979221

[30] Cincotta AH Meier AH. Bromocriptine (Ergoset) reduces body weight and improves glucose tolerance in obese subjects. Diabetes care (1996) 19(6):667–70. 10.2337/diacare.19.6.667 8725871

[31] Johnston R Uthman O Cummins E Clar C Royle P Colquitt J Canagliflozin, dapagliflozin and empagliflozin monotherapy for treating type 2 diabetes: systematic review and economic evaluation. Health Technol Assess (Winchester, England) (2017) 21(2):1–218. 10.3310/hta21020 PMC5292646 28105986

[32] Richardson JR Pipkin JA O'Dell LE Nazarian A. Insulin resistant rats display enhanced rewarding effects of nicotine. Drug and alcohol dependence (2014) 140:205–7. 10.1016/j.drugalcdep.2014.03.028 24774962 PMC10292761

[33] Klausen MK Thomsen M Wortwein G Fink-Jensen A. The role of glucagon-like peptide 1 (GLP-1) in addictive disorders. Br J Pharmacol (2022) 179(4):625–41. 10.1111/bph.15677 34532853 PMC8820218

[34] Cruz B Flores RJ Uribe KP Espinoza EJ Spencer CT Serafine KM Insulin modulates the strong reinforcing effects of nicotine and changes in insulin biomarkers in a rodent model of diabetes. Neuropsychopharmacol official Publ Am Coll Neuropsychopharmacol (2019) 44(6):1141–51. 10.1038/s41386-018-0306-3 PMC6461916 30647447

